# Total Hip Arthroplasty in Ankylosing Spondylitis: A Case Report of Ankylosed Hip

**DOI:** 10.7759/cureus.51619

**Published:** 2024-01-03

**Authors:** Kevin Kawde, Khizar K Khan, Gajanan Pisulkar, Shounak Taywade, Adarsh Jayasoorya

**Affiliations:** 1 Orthopedics, Jawaharlal Nehru Medical College, Wardha, IND

**Keywords:** rehabilitation, total hip arthroplasty, hip joint, osteoarthritis, ankylosing spondylitis

## Abstract

Ankylosing spondylitis (AS) is a chronic inflammatory arthritic disease that primarily affects the axial skeleton, and its association with the secondary development of osteoarthritis (OA) in peripheral joints, particularly the hips, is increasingly recognized. This case report elucidates the diagnostic and therapeutic challenges encountered in a patient with bilateral hip osteoarthritis secondary to AS. The patient's medical history included AS and a failed attempt at core decompression of the left hip joint. The patient was managed with total hip arthroplasty (THA) on the left side due to persistent symptoms. Total hip arthroplasty on the left side involved a meticulous surgical approach, addressing the unique challenges posed by underlying ankylosis. The procedure was conducted uneventfully, with the implantation of a modular femoral head, uncemented femoral stem, and modular shell. Postoperatively, the patient experienced significant pain relief and improved functionality. Successful rehabilitation and management were integral to the overall positive outcome. This case report highlights the complex interplay between AS and hip osteoarthritis, emphasizing the importance of tailored diagnostic and therapeutic strategies. Successful total hip arthroplasty in the setting of AS-related hip osteoarthritis suggests that joint replacement can be effective, but ongoing research is necessary to optimize surgical planning and long-term outcomes in this patient population.

## Introduction

Ankylosing spondylitis (AS) is a chronic inflammatory disease primarily affecting the axial skeleton, characterized by sacroiliitis, spondylitis, and enthesis. Although the hallmark features of AS involve the spine, extra-articular manifestations such as peripheral joint involvement are increasingly recognized as contributors to the overall disease burden [1]. One significant complication associated with AS is the development of secondary osteoarthritis (OA) in peripheral joints, particularly the hip joints. The prevalence of hip involvement in AS patients ranges from 25% to 50%, significantly impacting the patient's quality of life and functional capacity [2,3]. The pathogenesis of hip osteoarthritis in AS is multifactorial and involves chronic inflammation, biomechanical stress, and structural changes that collectively contribute to articular cartilage damage and degeneration [4].

Diagnosing and managing osteoarthritis in the context of AS pose challenges due to the overlapping clinical manifestations and the potential for the delayed recognition of osteoarthritis-related symptoms. Additionally, the impact of AS on the response to surgical interventions, such as total hip arthroplasty (THA), further complicates the management of these dual pathologies [5]. This case report aims to contribute to the understanding of the complex interplay between AS and hip osteoarthritis, shedding light on the diagnostic variations and therapeutic considerations in such cases. By presenting a detailed account of the clinical presentation, diagnostic workup, and surgical intervention in a patient with bilateral hip osteoarthritis secondary to AS, this report seeks to enhance the existing knowledge base and guide future clinical practice.

## Case presentation

A 45-year-old male presented with a chief complaint of pain and restriction of movements in both hips since 2013. The patient reported an insidious onset and gradual progression of pain, describing it as mild-to-moderate intensity. The pain is exacerbated with movement, walking, sitting down, and getting up, with no significant diurnal variation. Notably, there was a history of operative intervention in the form of core decompression of the left hip joint in 2015. However, the patient noted an increase in pain and restriction of movements following the procedure.

There was no history of trauma; loss of consciousness; ear, nose, and throat (ENT) bleeding; fever; cough; cold; or steroid intake. The patient had a known history of tuberculosis in the left hip. Medical history revealed no significant comorbidities such as diabetes, hypertension, asthma, or allergies. Additionally, no remarkable past medical or family history was reported, and the patient's bowel and bladder habits were normal.

Physical examination revealed an antalgic gait. The left hip was fixed in 20-degree external rotation and 20-degree flexion, whereas the right hip was fixed in 15-degree flexion and 10-degree external rotation. Lumbar lordosis was lost, and bilateral knee and ankle ranges of motion were full and painless. Active toe movements were present, with intact distal circulation and no neurodeficit.

**Figure 1:**
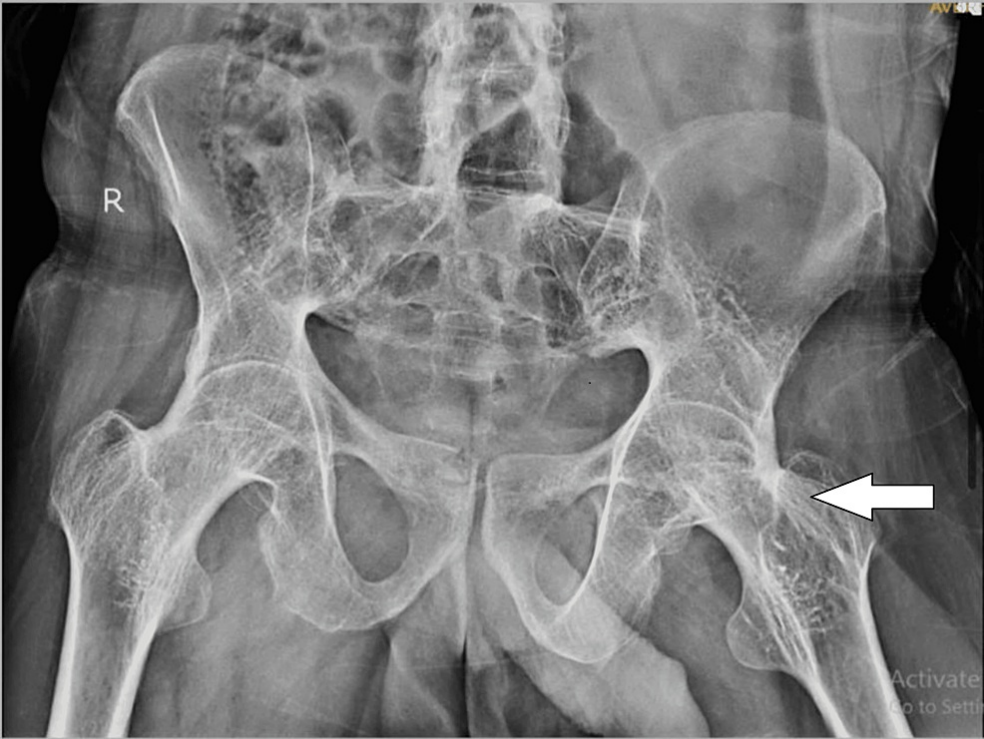
Pelvis X-ray with bilateral hip AP view s/o osteoarthritis of the bilateral hip secondary to ankylosing spondylitis AP, anteroposterior; s/o, suggestive of

**Figure 2:**
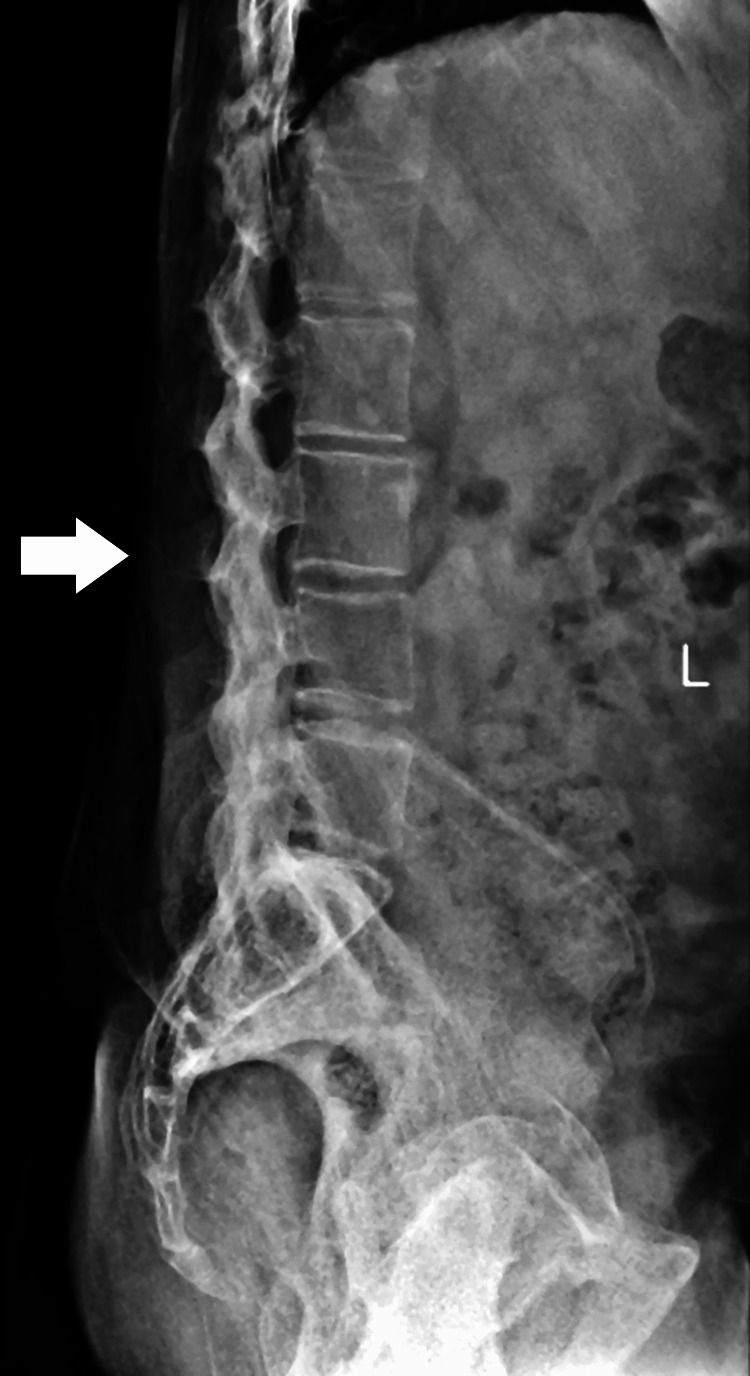
Lumbo-sacral spine X-ray AP view suggestive of ankylosed spine AP: anteroposterior

**Figure 3:**
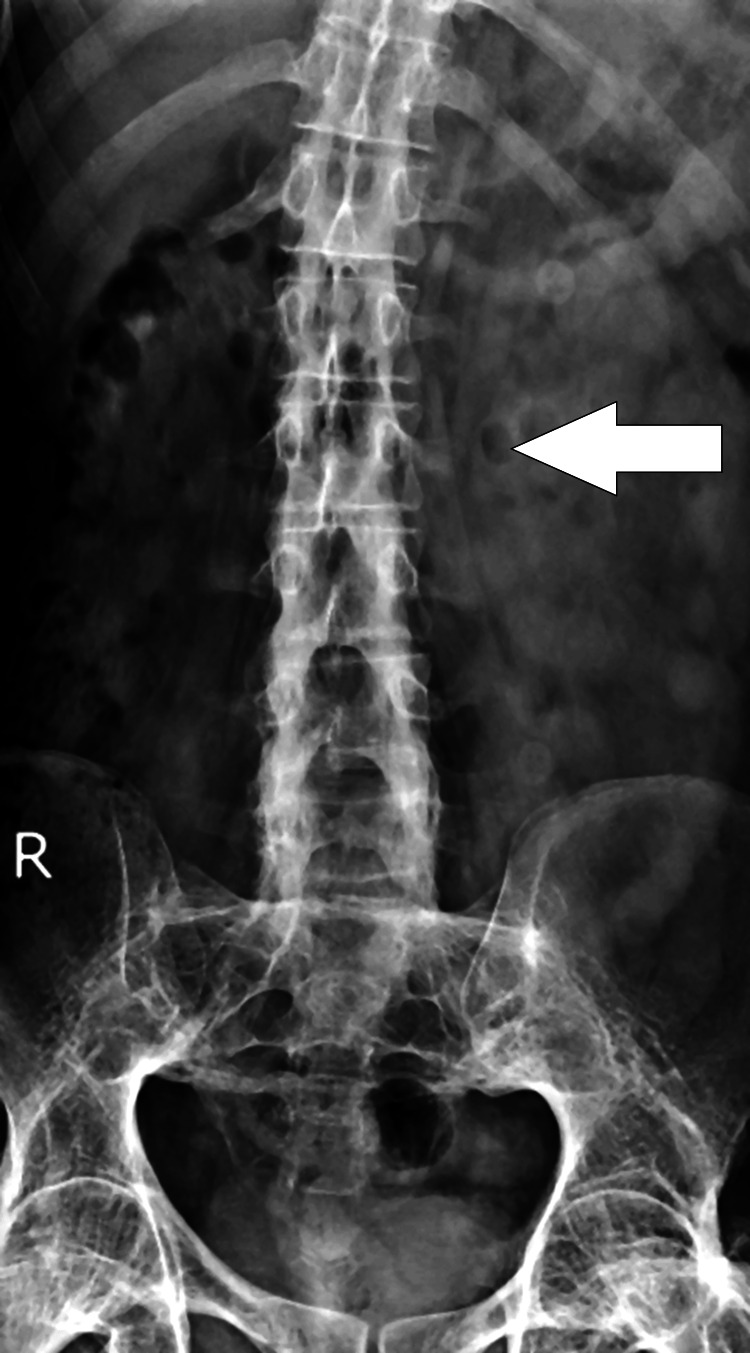
Lumbo-sacral spine X-ray AP view suggestive of ankylosed spine AP: anteroposterior

On radiological examination, the pelvis X-ray with bilateral hip anteroposterior (AP) view was suggestive of bony ankylosis of the bilateral hip (Figure 1), and the lumbo-sacral spine X-ray AP view showed features of ankylosis, that is, bamboo spine (Figures 2, 3). Considering the persistent symptoms, limited mobility, and severity of the disease, a decision was made to proceed with total hip arthroplasty of the left hip. Taking into account the extensive nature of the surgery, it was decided to operate the unilateral hip in one sitting.

**Figure 4:**
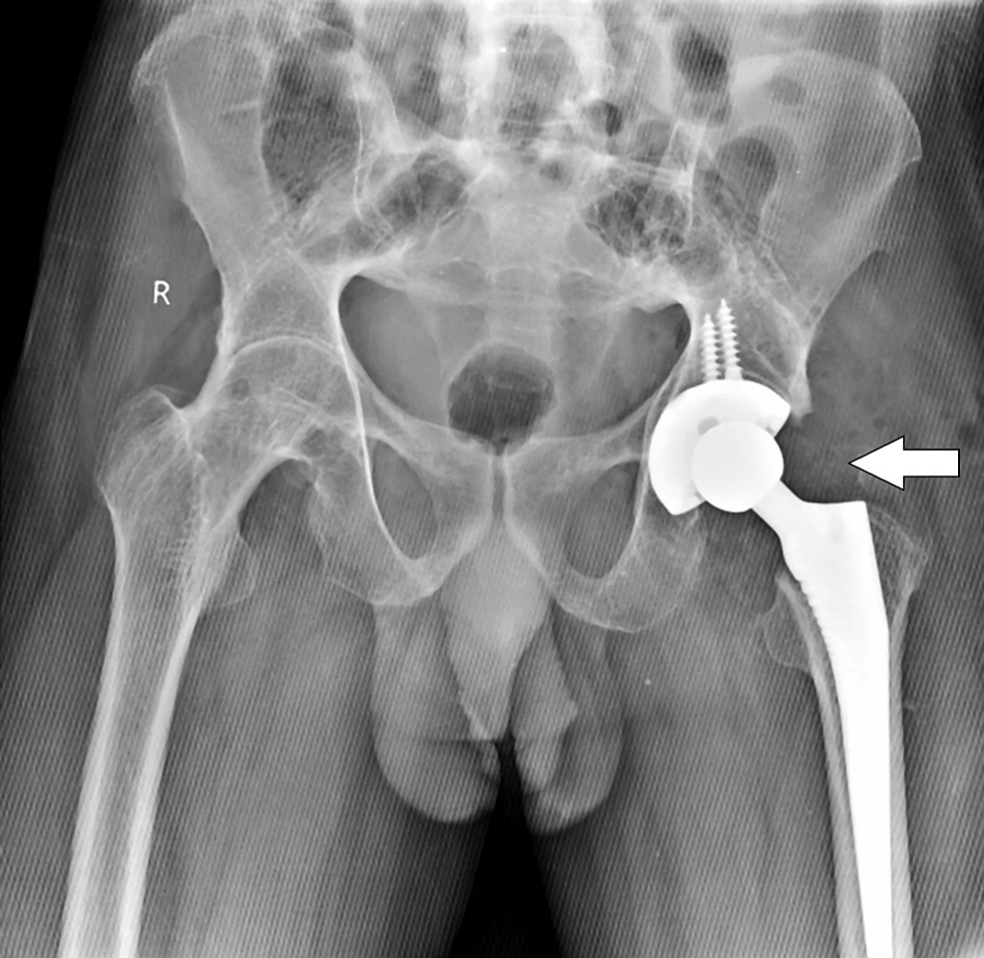
Pelvis X-ray with bilateral hips: postoperative X-ray of the operated case of total hip arthroplasty on the left side with implant in situ

During the operative procedure, conducted under spinal anesthesia, the following steps were taken: Through the posterolateral approach, soft tissue dissection was done, and external rotators were tagged and detached. Plain for femoral neck cut was marked after reaching the inferior neck and locating the pubofemoral arch and lesser trochanter. The markings for neck cut were also confirmed under C-arm due to disturbed anatomy; this spared the requirement of trochanteric osteotomy. Acetabular cup positioning and reaming were aided by the placement of superolateral bone spikes. The femoral head was removed in a piecemeal fashion as the femoral head was fused with acetabulum due to bony ankyloses. Sequential reaming was done under intraoperative C-arm guidance to locate the original joint line and the foveal structures. Multihole trabecular metal acetabular cup was pressed fit in the acetabulum and fixed with two screws. A porous coated tapered stem of size 3/135 mm was selected for femoral reconstruction. As there were multiple contractures, soft tissue release was done to achieve the reduction even with the smallest femoral head, that is, 28 mm (Figure 4).

After the surgical procedure, the patient experienced a smooth recovery without any notable incidents. The stability and range of motion in the hip were assessed and confirmed and satisfactory. The patient received meticulous care, including applying a sterile dressing and a crepe bandage. A comprehensive postoperative care and rehabilitation plan were instituted. Scheduled follow-up visits were established for one year to diligently track the recovery progress and promptly address any potential complications.

## Discussion

The presented case highlights the intricate interplay between AS and the development of secondary osteoarthritis (OA) in the bilateral hip joints, underscoring the challenges in diagnosis and management. The association between AS and peripheral joint involvement, particularly in the hips, has been extensively documented [1,3]. In this case, the patient's 10-year history of insidious-onset, progressively worsening hip pain aligned with the established clinical profile of hip involvement in AS [2]. The diagnostic journey in AS-related hip OA often involves a multidisciplinary approach, considering the overlapping symptoms with primary hip OA and the unique challenges posed by the underlying inflammatory nature of AS [6]. The patient underwent core decompression of the left hip joint in 2015, a procedure commonly employed in early-stage hip OA. However, the reported worsening of symptoms postoperatively emphasizes the need for nuanced decision-making in the context of AS-associated OA, where inflammatory processes may compromise the outcomes of joint-preserving procedures [7].

In this case, the total hip arthroplasty (THA) performed on the left side represents a crucial intervention, offering significant pain relief and improved function. However, the surgical management of hip OA in the AS setting is complex. The altered biomechanics and potential for spinal involvement in AS patients may influence surgical planning and outcomes [8]. The successful THA described in this report aligns with existing literature emphasizing the efficacy of joint replacement in advanced cases of AS-related hip OA [9]. While THA is a well-established intervention, the influence of AS on postoperative outcomes remains a subject of ongoing research. Factors such as disease activity, spinal involvement, and extra-articular manifestations may impact the long-term success of THA in AS patients [10]. Therefore, long-term follow-up and careful monitoring of the patient's overall disease activity and joint function are imperative.

## Conclusions

In conclusion, the successful management of this case highlights the importance of adapting therapeutic strategies to the unique challenges posed by ankylosing spondylitis-associated hip osteoarthritis. Further research and collaborative efforts are warranted to refine diagnostic approaches, optimize surgical interventions, and enhance the long-term outcomes for individuals navigating the complex interplay between ankylosing spondylitis and hip osteoarthritis.
